# TCRBinder: Unified pre-trained language model with paired-chain synergy for predicting T-cell receptor binding specificity

**DOI:** 10.1371/journal.pcbi.1014396

**Published:** 2026-06-22

**Authors:** Weihe Dong, Qiang Yang, Long Xu, Xiaokun Li, Kuanquan Wang, Suyu Dong, Gongning Luo, Xianyu Zhang, Tiansong Yang, Xin Gao, Guohua Wang

**Affiliations:** 1 Faculty of Computing, Harbin Institute of Technology, Harbin, China; 2 Zhengzhou Advanced Research Institute, Harbin Institute of Technology, Zhengzhou, China; 3 School of Computer and Big Data, Heilongjiang University, Harbin, China; 4 Postdoctoral Program of Heilongjiang Hengxun Technology Co., Ltd., Harbin, China; 5 Shandong Hengxun Technology Co., Ltd., Qingdao, China; 6 Computer Science Program, Computer, Electrical and Mathematical Sciences and Engineering Division, King Abdullah University of Science and Technology (KAUST), Thuwal, Kingdom of Saudi Arabia; 7 Center of Excellence for Smart Health (KCSH), King Abdullah University of Science and Technology (KAUST), Thuwal, Kingdom of Saudi Arabia; 8 Center of Excellence on Generative AI, King Abdullah University of Science and Technology (KAUST), Thuwal, Kingdom of Saudi Arabia; 9 Department of Breast Surgery, Harbin Medical University Cancer Hospital, Harbin, China; 10 Department of Rehabilitation, The First Affiliated Hospital of Heilongjiang University of Traditional Chinese Medicine, Harbin, China; Universita degli Studi di Torino, ITALY

## Abstract

Deciphering how human T cells recognise peptide-HLA (pHLA) complexes underpins next-generation vaccines and personalised immunotherapies, yet extreme sequence diversity and paired-chains interdependence still hamper reliable *in silico* prediction of T-cell receptor (TCR) specificity. To overcome these hurdles, we built TCRBinder, a paired-chain-aware deep model with a multi-branch encoder that routes each molecular component through dedicated transformer-based modules to capture contextual signals in both HLA pseudo-sequences and antigenic peptides while simultaneously processing the TCR α and β chains. This design captures the synergistic interaction between paired chains to emulate peptide-HLA-TCR (PHT) interactions and expose residue-level contact motifs. Across PHT and peptide-TCR (pTCR) benchmarks, the model delivered state-of-the-art performance (AUC-ROC = 0.911, AUPR = 0.791 for the PHT task) and remained superior on multiple independent datasets. We tracked the dynamics of clonal expansion and, in a large SARS-CoV-2 repertoire containing completely unseen peptides, improved the AUC-ROC by up to 16.3% over the leading alternatives. Moreover, TCRBinder provided mechanistic insights by pinpointing contact hotspots and quantifying residue contributions to binding probability. These capabilities position TCRBinder as a versatile tool for rational antigen discovery, immunotherapy stratification, and neoantigen vaccine design.

## Author summary

T-cell receptors (TCRs) play a central role in adaptive immunity by recognizing peptide-HLA complexes, but predicting their binding specificity has remained a formidable challenge due to sequence diversity and the cooperative action of α and β chains. Existing computational methods often rely on limited CDR3β information or single-chain inputs, which restrict predictive accuracy and generalization. To address this, we developed TCRBinder, a unified deep learning framework that processes full-length paired TCR chains together with peptide and HLA sequences. The model combines pre-trained encoders, ESM2 and Roformer, with a multi-fusion convolutional network to capture both global semantic context and local interaction motifs. Across benchmark and independent datasets, TCRBinder consistently surpasses leading tools, demonstrating state-of-the-art performance and robust transferability to unseen epitopes and external clinical repertoires. Importantly, its predictions correlate with *in vivo* clonal expansion and highlight key residues that shape recognition, providing interpretable insights into molecular binding mechanisms. By integrating predictive accuracy with biological relevance, TCRBinder offers a versatile platform for antigen discovery, immunotherapy design, and the development of personalized vaccines.

## Introduction

T-cell receptor (TCR) based immunotherapies, including checkpoint inhibitors and engineered T cell therapies, have revolutionized cancer treatment by leveraging neoantigens to trigger potent immune responses [1–3]. The effectiveness of these therapies relies on the precise recognition of antigenic peptides presented by human leukocyte antigen (HLA) molecules, which initiate T cell activation and tumor cell elimination [4–6]. However, the limited response rates in many patients stem from challenges in identifying neoantigens that elicit strong TCR binding [7–9]. In the cancer immunity cycle, TCR recognition of antigenic peptide-HLA (pHLA) complexes is a critical step for T cell activation and effector function [10,11]. Experimental techniques, including tetramer staining and high-throughput sorting, remain labor intensive and time costly, underscoring the need for scalable computational frameworks to predict TCR binding specificity and facilitate individual immunotherapy [12–14].

The binding of TCRs to pHLA complexes is orchestrated by the synergistic interplay of the TCR α and β chains, with complementarity-determining region 3 (CDR3) serving as the primary determinant of specificity [15,16]. The human TCR repertoire exhibits immense diversity, with theoretical estimates suggesting that V(D)J recombination can generate on the order of 10^18^ distinct α and β TCRs [17,18]. This vast sequence variability, coupled with the extensive polymorphism of HLA molecules, poses significant hurdles for computational modeling in antigen presentation and TCR recognition [19,20]. To achieve precise pHLA-TCR (PHT) binding prediction, models must capture the cooperative dynamics of TCR α and β chains and their nuanced interactions with diverse pHLA complexes, necessitating advanced deep learning solutions.

Current computational approaches for pHLA and peptide-TCR (pTCR) binding prediction have made notable progress but face critical limitations [21]. Tools like HLAIImaster [22], MHLAPre [23], NetMHCpan4.0 [24] and MHCflurry [25] excel in predicting pHLA binding using deep neural networks, while pTCR prediction models, such as ImRex [26], TEIM [27], and TCR-AI [28], focus on peptide-CDR3 interactions, often neglecting HLA context or TCR chain synergy. Previous methods like pMTnet [21] and THLANet [29] aim to model PHT relationships but are constrained by single-chain TCR representations or specific HLA alleles, limiting their ability to address paired chain dynamics. The scarcity of validated binding data further exacerbates these challenges, highlighting the need for a unified model that integrates comprehensive interaction data to improve predictive performance.

To address these challenges, we introduce TCRBinder, a paired chain aware deep learning framework that harnesses pre-trained protein language models to predict PHT binding specificity with improved accuracy. TCRBinder employs a multi-branch encoder architecture to independently process TCR α and β chains, HLA pseudo-sequences, and antigen peptides, utilizing ESM2 and Roformer modules to learn rich, modality-specific representations. These high-dimensional representations are then integrated via a multi-branch fusion block with Multi-Fusion Convolutional Neural Networks (MFCNNs). This unified approach enables the simultaneous prediction of PHT and pTCR binding specificity, providing interpretable insights into residue-level interactions and offering a versatile platform for neoantigen discovery and immunotherapy design.

TCRBinder outperforms existing methods in predicting PHT and pTCR binding specificities, delivering three transformative contributions. First, its unified architecture facilitates integrated prediction of PHT and pTCR interactions, enabling a comprehensive evaluation of neoantigen immunogenicity that enhances understanding of T cell activation. This flexibility supports multiple tasks, including pTCR and PHT binding, within a single framework. Second, we employ pre-training on large-scale protein datasets and multitask fine-tuning on curated TCR repositories, such as VDJdb [30] and IEDB [31], to overcome data scarcity and ensure robustness across diverse HLA alleles. Third, extensive validation on independent datasets demonstrates an improvement of more than 16.3% in Area Under the Receiver Operating Characteristic Curve (AUC-ROC) over state-of-the-art models, with saliency maps elucidating key residue interactions. These advancements position TCRBinder to streamline neoantigen screening, advance TCR-engineered therapies, and provide molecular insights into immune recognition, laying the groundwork for precision immunotherapy.

## Results

### TCRBinder overview

**Fig 1:**
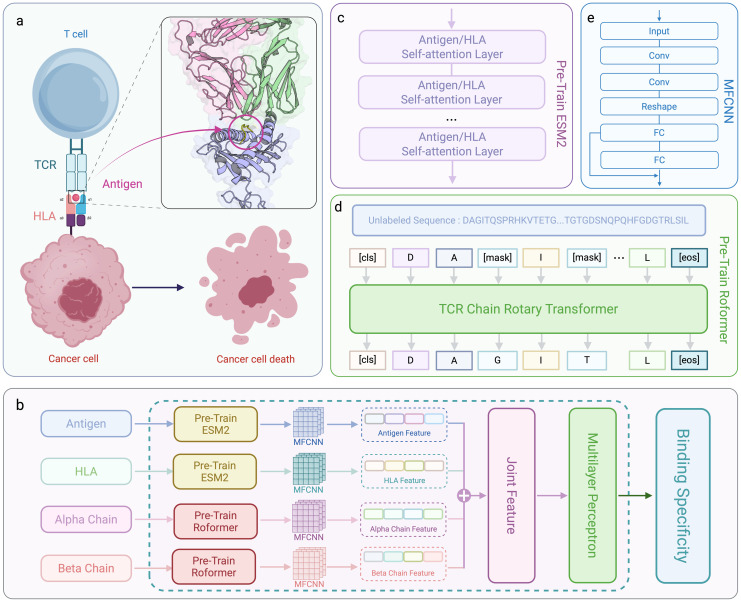
Overview of TCRBinder and its components. **(a)** Schematic of T cell-mediated cytotoxicity. A T cell utilizes its T cell receptor (TCR) to recognize an antigen presented by an HLA molecule on a cancer cell. This specific interaction, illustrated in the magnified view of the PHT complex, is the critical event that triggers the destruction of the cancer cell. **(b)** The framework of the PHT binding specificity predictor. It takes the antigen, HLA, and TCR α and β chain sequences as input. Each sequence is encoded by passing through a pre-trained encoder (ESM2 for antigen and HLA; Roformer for TCR chains) and a Multi-Fusion Convolutional Neural Network (MFCNN) feature extractor. A multilayer perceptron model (MLP) finally fuses the features from all four sequences to predict the binding specificity. **(c)** Internal architecture of the antigen and HLA encoders. The ESM2-based models for both antigen and HLA consist of multiple stacked self-attention layers, which are pre-trained on large protein sequence databases to learn generalizable representations. **(d)** Schematic of the self-supervised pre-training of the TCR Roformer. Unlabeled TCR chain sequences are used to pre-train the TCR Roformer via masked language modeling. The model is trained to predict the identity of masked tokens, thereby learning generalizable representations of TCR sequences. **(e)** The architecture of the MFCNN. The MFCNN module integrates convolutional and fully connected layers to extract high-level biological features from the encoder outputs. Created in BioRender. Li, X. (2026) https://BioRender.com/px2pbv5.

T-cell receptors (TCRs) play a pivotal role in adaptive immunity by recognizing peptide antigens presented on human leukocyte antigen (HLA) molecules, enabling T cells to distinguish self from non-self and target aberrant cells, such as those in cancer (Fig 1a). This recognition process involves intricate interactions between the TCR α and β chains, the antigenic peptide, and the HLA molecule, culminating in T-cell activation and effector functions like cytotoxicity against tumor cells [32–34]. To computationally predict TCR binding specificity with high fidelity, we developed TCRBinder, a unified pre-trained language model that incorporates paired chain synergy, departing from conventional methods that focus narrowly on CDR3β regions. By modeling full-length TCR α and β chains alongside peptide and HLA sequences, TCRBinder captures the biophysical nuances of ternary complex formation, including long-range dependencies and cross-chain interactions.

The core of TCRBinder comprises four specialized encoders pre-trained on vast immunological datasets to learn modality-specific embeddings (Fig 1b). The α-Roformer and β-Roformer, transformer-based encoders with rotary positional embedding, are pre-trained on millions of unpaired full-length TCR sequences using masked language modeling. This allows the model to learn robust chain-specific contextual representations, which are subsequently integrated to capture the synergistic interaction between paired chains during downstream tasks (Fig 1d). In parallel, the Antigen-ESM2 and HLA-ESM2 encoders leverage the large-scale protein language model ESM2 [35], which is pre-trained using UniRef50 protein sequences, employing stacked self-attention layers to extract residue-level dependencies and polymorphic patterns from antigenic peptides and HLA pseudo-sequences (Fig 1c). This pre-training strategy ensures that each encoder specializes in its respective domain, with the peptide encoder capturing positional motifs, the HLA encoder modeling allele-specific binding grooves, and the TCR encoder capturing long-range dependencies and sequence motifs across full-length TCR chains, which together yield robust and transferable features for binding prediction.

These specialized embedding representations are integrated via a multi-branch fusion block, which utilizes multi-scale CNN layers to process spatially aligned features, followed by reshaping and fully connected layers to learn high-level biological features. Subsequently, these captured features are concatenated and passed through a multilayer perceptron (MLP) network to predict PHT binding specificity (Fig 1b and 1e). Supervised training on a curated dataset that comprises 12,827 experimentally validated PHT binders together with a fivefold larger set of approximately 65,000 *in silico* negative decoys (**Methods**) collectively optimizes the model for accurate discrimination. This architecture facilitates cross-modal learning of interaction determinants and enhances generalization to unseen epitopes and donor repositories, as it outperforms CDR3β-centric baselines by leveraging holistic chain-level information and reducing overfitting to sparse binding data.

### Evaluation of binding prediction performance

**Fig 2:**
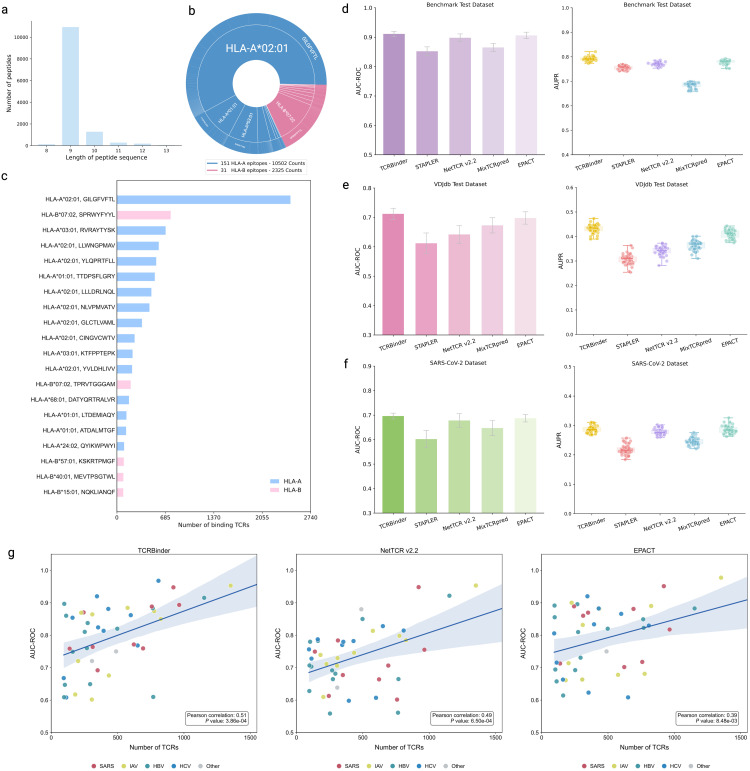
Dataset characteristics and performance benchmarking. **(a)** Frequency distribution of epitope peptide lengths in the benchmark dataset. **(b)** Distribution of TCRs recognizing epitopes restricted to different HLA alleles in the benchmark test dataset. **(c)** Number of binding αβTCRs for the top 20 pHLAs with the most experimentally validated TCRs. **(d)** AUC-ROC and AUPR of TCRBinder and state-of-the-art methods on the benchmark test dataset. **(e)** AUC-ROC and AUPR of TCRBinder and state-of-the-art methods on the VDJdb test dataset. **(f)** AUC-ROC and AUPR of TCRBinder and state-of-the-art methods on the SARS-CoV-2 dataset. **(g)** Correlation analysis between epitope-associated TCR counts and AUC-ROC for the TCRBinder, NetTCR v2.2, and EPACT on the benchmark test set. Created in BioRender. Li, X. (2026) https://BioRender.com/px2pbv5.

PHT binding specificity plays a pivotal role in expediting immunotherapies that pinpoint robust T-cell responses to emerging threats or cancers. To rigorously benchmark TCRBinder’s proficiency in forecasting PHT binding specificity, we created a robust repository of full-sequence PHT engagements. This corpus encompasses vast TCR recognized antigens ranging from 8 to 13 residues (Fig 2a) and common HLA alleles (Fig 2b). As observed, a handful of privileged pHLA complexes monopolize the binding landscape, which is exemplified by HLA-A*02:01, GILGFVFTL and HLA-B*07:02, SPRWYFYYL that engage myriad TCR variants and epitomize immunodominant biases (Fig 2c). Given that TCR interrogation hinges on prior peptide stabilization within HLA pockets, we scrutinized TCRBinder’s aptitude for predicting PHT binding specificity against leading comparators, including STAPLER [36], NetTCR v2.2 [37], MixTCRpred [38], and EPACT [39]. This evaluation utilizes a dataset of 12,827 PHT complexes spanning 174 distinct peptides and 23 HLA subtypes (**Methods**).

We employ gold-standard evaluation metrics, including the Area Under the Receiver Operating Characteristic Curve (AUC-ROC) and the Area Under the Precision-Recall Curve (AUPR), Macro-averaged AUC 0.1 (AUC0.1) [40], F1-Score, and accuracy to comprehensively assess the model performance (as shown in S1 Appendix Section A). To ensure fair and consistent evaluation, we compared our model with state-of-the-art baseline methods (as shown in S1 Appendix Section B) under identical datasets and training strategies. TCRBinder excelled with an AUC-ROC of 0.911, an AUC0.1 of 0.694, and an AUPR of 0.791 on the evaluation partition (Fig 2d and S1 Figa), outperforming STAPLER (AUC-ROC 0.852, AUC0.1 0.561, AUPR 0.765), NetTCR v2.2 (0.898, 0.673, 0.781), MixTCRpred (0.865, 0.582, 0.693), and EPACT (0.906, 0.686, 0.785). Additionally, TCRBinder maintained strong accuracy, precision, and F1-score (S1 Fig and S1 Table), attesting to the stability and reliability of its predictive framework. Broadening the lens to pTCR dynamics, the framework sustained dominance, yielding an AUC-ROC of 0.886 and AUPR of 0.745 (S2 Fig), underscoring its versatility in unraveling intricate recognition hierarchies.

To understand the limitations imposed by data sparsity, we analyzed the correlation between the number of training TCRs per epitope and model performance (AUC-ROC) (Fig 2g). As expected, all models exhibited a significant positive correlation, confirming that predictions generally improve with increased data availability. Notably, TCRBinder showed the highest Pearson correlation coefficient (r = 0.51), compared to NetTCR v2.2 (r = 0.49) and EPACT (r = 0.39). This strong correlation demonstrates TCRBinder’s superior capacity to leverage available training data for performance gains.

**Fig 3:**
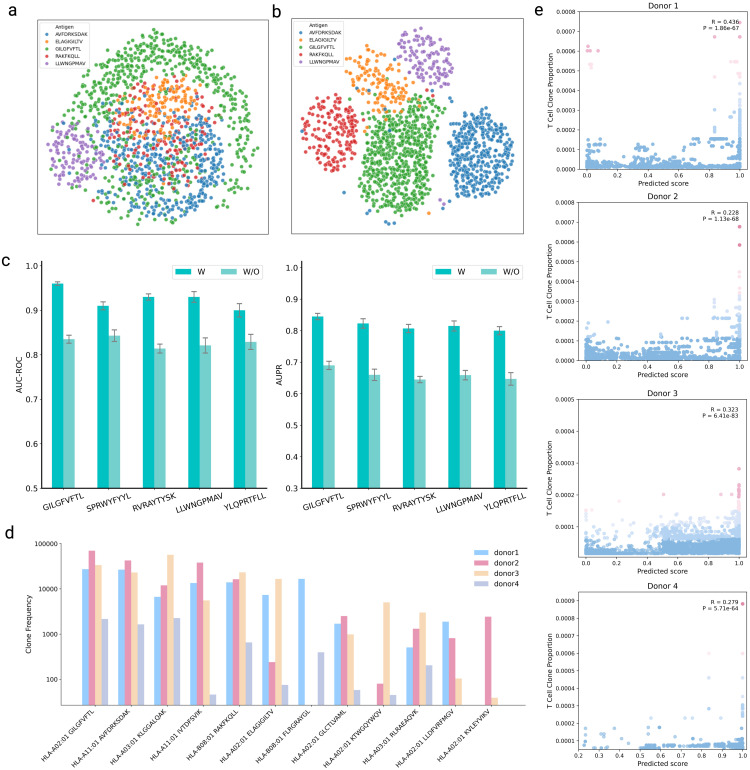
TCRBinder captures Antigen-Specific features and correlates with clonal expansion. **(a-b)** UMAP projections of TCR sequence embeddings, obtained from the randomly initialized Roformer (a) and the pre-trained Roformer **(b)**. **(c)** Comparison of AUC-ROC and AUPR scores between the model with pre-training (W) and the model without pre-training (W/O) across five representative antigenic epitopes. **(d)** Clone-frequency distributions for pHLAs across four donors profiled with 10x Genomics single-cell Peptide-HLA capture. **(e)** In the 10x Genomics Chromium immune-profiling dataset, the proportion of T-cell clones shows a significant positive correlation with the TCRBinder-predicted binding score for the corresponding pHLA across all four donors. Created in BioRender. Li, X. (2026) https://BioRender.com/px2pbv5.

To determine whether the pre-training stage furnishes the representational structure needed to meet this challenge, we first inspected the latent space learned by TCRBinder. Pre-training markedly improves representation quality. Using UMAP to visualize embeddings, the untrained model (Fig 3a) yields scattered, poorly separable features, whereas the pre-trained model (Fig 3b) forms compact, epitope specific clusters. This clearer structure indicates larger inter epitope differences are being captured and that pre-training strengthens the model’s ability to extract discriminative features for epitope recognition.

### Generalization experiments

We conducted two evaluation scenarios to assess the generalization ability of TCRBinder in predicting PHT binding specificity, designed to mimic real world applications. The first scenario focused on predicting binding TCRs for previously unseen epitopes, while the second scenario evaluated the ability to predict the binding specificity of a distinct TCR population from the VDJdb database [30]. Unlike methods that rely solely on single-chain CDR3 regions, TCRBinder was trained on full-length paired TCR α and β sequences, allowing it to capture a more comprehensive set of structural and sequence determinants that govern pHLA recognition.

In the unseen-epitope setting (as shown in S3 Fig), TCRBinder achieved an AUC-ROC of 0.599 and an AUPR of 0.225, substantially outperforming the comparative baseline methods STAPLER [36], NetTCR v2.2 [37], MixTCRpred [38], and EPACT [39]. To examine whether performance in the unseen-epitope setting could be influenced by HLA-related or peptide–HLA-related shortcuts, we first evaluated a fixed-HLA control, in which HLA identity was held constant during negative construction. Under this setting, TCRBinder achieved an AUC-ROC of 0.597 and an AUPR of 0.224. Because this control does not fully exclude peptide–HLA confounding, we further introduced a stricter fixed-pHLA control, in which both the peptide and HLA were kept unchanged and negative pairs were generated only by replacing the TCR. Under this more stringent setting, TCRBinder achieved an AUC-ROC of 0.572 and an AUPR of 0.206. Although performance decreased under this more stringent control, it remained above chance, suggesting that the prediction is not explained solely by HLA identity or peptide–HLA pairing. Beyond these area-based metrics, classification performance also demonstrated the model’s strength, with consistently high accuracy, precision, and F1-score (S4 Fig and S2 Table). These results indicated that TCRBinder can effectively generalize to novel antigenic targets outside its training distribution, a capability essential for applications such as emerging pathogen surveillance and cancer neoantigen discovery. In the second setting, using unique PHT pairs from the VDJdb database, TCRBinder maintained strong predictive performance with an AUC-ROC of 0.712 and an AUPR of 0.430 (Fig 2e). The model again exhibited high accuracy, precision, and F1-score across this independent evaluation (S1 Fig and S3 Table), demonstrating its robustness across different data sources and TCR repertoires. The ability to sustain performance in the VDJdb evaluation suggests that TCRBinder captures generalizable sequence-structure-specificity relationships that are transferable across populations, experimental platforms, and epitope repertoires.

These generalization experiments highlight TCRBinder’s ability to handle both epitope level novelty and repertoire level diversity. Its use of full-length paired TCR α and β chains enables richer feature representation than single-chain approaches, enhancing recognition of binding determinants that remain consistent across unseen peptides and distinct TCR populations. This robustness, coupled with superior classification metrics compared to other advanced methods, underscores its suitability for real-world deployment in immunotherapy development, epitope prioritization, and immune repertoire monitoring.

To further contextualize TCRBinder’s performance within recent community benchmarks, we compared our sequence-based approach against three AlphaFold-based baselines on the corresponding IMMREP25 [41] allele-specific benchmark subsets for HLA-A02:01 and HLA-B40:01 (S5 Fig). These analyses used the same peptide–TCR pairs as those in the respective benchmark subsets. The compared AlphaFold-based baselines were AF3-TCRdock [42], AF2-TCRdock [43], and AF2-TCRmodel2 [44]. Performance was summarized using per-peptide mean AUROC. Additional details on the benchmark composition, structure generation procedures, and model-derived ranking scores used for classification are provided in S1 Appendix Section C. Under this matched evaluation setting, TCRBinder outperformed the AlphaFold-based baselines on HLA-A02:01, whereas the AlphaFold-based methods retained an advantage on HLA-B40:01.

### SARS-CoV-2-responsive PHT dataset

To further investigate the application potential of TCRBinder, we evaluated its performance on a clinically relevant, external PHT recognition dataset. This dataset was constructed using samples of SARS-CoV-2-responsive T cells [45], allowing for a robust assessment of the model’s ability to identify antigen-specific TCRs from a complex repertoire. We benchmarked TCRBinder against several other state-of-the-art pan-specific models, including STAPLER [36], NetTCR v2.2 [37], MixTCRpred [38], and EPACT [39], to assess its predictive power in a direct comparison. TCRBinder delivered a remarkable and consistently superior performance across all metrics. Specifically, it achieved the AUC-ROC of approximately 0.696 and also achieved the AUPR of around 0.302 (Fig 2f). TCRBinder again exhibited high accuracy, precision, and F1-score across this independent evaluation (S4 Fig and S4 Table).

TCRBinder’s superior ability to identify TCRs responsive to viral epitopes is attributable to its core design choices, including its use of full-length paired TCR α and β chain sequences, pre-training-derived priors, and position-aware encoding. Together, these features allow the model to capture complex inter-chain complementarity and contextual information that extends far beyond the TCR β chain CDR3 region alone. In practical terms, these results indicate that TCRBinder can effectively generalize from training distributions to real-world clinical repertoires exposed to diverse SARS-CoV-2 antigens, maintaining robust discrimination across individuals and targets. This thereby supports its powerful application in immune monitoring and in enabling a rapid response to emerging viral variants.

### Ablation experiments

To quantify the contribution of different architectural components and encoding strategies to TCRBinder, we systematically constructed a set of ablated variants: (1) without the TCR α chain (tested in both CDR3-only and full β-chain configurations); (2) without HLA information, omitting the HLA pseudo-sequence branch; (3) without MFCNN, in which the multi-scale convolution architecture was replaced by a standard Multilayer Perceptron (MLP) fusion block; and (4) variants replacing our custom TCR-Roformer with existing encoders (TCR-ESM2 and TCR-Lang-Paired [46]).

As shown in S5 Table, we evaluated the robustness of these results by reporting the mean and standard deviation from independent experimental runs. Statistical significance between the full TCRBinder model and each ablated variant was evaluated using a two-sided Welch’s t-test on AUC-ROC. To account for multiple comparisons across the seven ablation tests, we applied the Bonferroni correction and used a Bonferroni-adjusted significance threshold ($\alpha_{adj}$=0.0071). Under this criterion, excluding HLA information resulted in a significant decrease in performance (AUC-ROC = 0.886 ± 0.015, $P=2.73\times 10^{-3}$), indicating that HLA context provides important constraints for accurate binding prediction even in the pan-specific setting. Likewise, removing the MFCNN module also reduced performance significantly (AUC-ROC = 0.898 ± 0.011, $P=4.25\times 10^{-3}$), supporting the contribution of this module to capturing local residue-level interaction patterns. In addition, paired-chain input remained advantageous over single-chain variants, with AUC-ROC decreasing to (0.871 ± 0.015) for the β-only model and to (0.862 ± 0.018) for the CDR3-only model, further supporting the contribution of combining the TCR α and β chains.

Regarding the encoding strategy, we validated the necessity of domain-specific pre-training by comparing TCRBinder against two representative baselines. Our custom TCR-Roformer encoder (AUC-ROC = 0.911 ± 0.009) outperformed both the general TCR-ESM2 (AUC-ROC = 0.892 ± 0.012) and the specialized TCR-Lang-Paired encoder (AUC-ROC = 0.901 ± 0.010). To further empirically verify the impact of Pre-training on epitope recognition, we trained a variant (Without Pre-training), where the model parameters were initialized randomly. As shown in S5 Table, this variant exhibited a substantial performance drop (AUC-ROC decreased to 0.795 ± 0.039, P < 0.001). Moreover, we performed the specificity prediction across five representative antigenic epitopes. As shown in Fig 3c, the pre-trained model (W) achieves higher AUC-ROC and AUPR scores across all tested epitopes compared to the model without pre-training (W/O). This further demonstrates that pre-training strengthens the model’s ability to extract discriminative features for epitope recognition.

### Prediction of TCRBinder highly correlates with T-Cell clonal expansion

To further validate our model, we assessed whether the PHT binding specificity predicted by TCRBinder reflects antigen-driven T-cell proliferation *in vivo*. We utilized a single-cell immune profiling dataset generated using the 10x Genomics Chromium platform [47], which profiled T cells from four healthy donors against a panel of 44 distinct pHLAs. Importantly, to ensure the rigor of this validation and exclude potential data leakage, all TCR clonotypes and interaction pairs from these four donors were strictly excluded from the model’s training dataset.

For each T cell, its antigen specificity was determined by counting the Unique Molecular Identifiers (UMIs) associated with each pHLA. A PHT binding event was classified as a positive interaction if its UMI count was 10 or greater, and cells with UMI counts below this threshold for a given pHLA were not considered in the analysis for that specific interaction (Fig 3d). TCRBinder then generated binding scores for each T-cell clonotype against all 44 pHLAs. To avoid compositional effects across donors, we used Spearman rank correlation to evaluate the association between the highest predicted binding score for each clone and its corresponding clonal expansion.

As shown in Fig 3e, we observed a consistent and statistically significant positive correlation between T-cell clone proportion and the predicted binding scores across all four donors. This finding strongly aligns with the biological expectation that TCRs with higher binding affinity are more prone to undergo clonal selection and expansion. The consistent trend across multiple donors confirms that our model’s predictions are biologically relevant and validates its ability to identify TCRs that are likely to mount a functional response. In summary, TCRBinder’s binding predictions capture meaningful determinants of antigen-specific clonal dynamics within human T-cell repertoires, underscoring the model’s strong relevance for adaptive immune response.

Key binding site analysis

**Fig 4:**
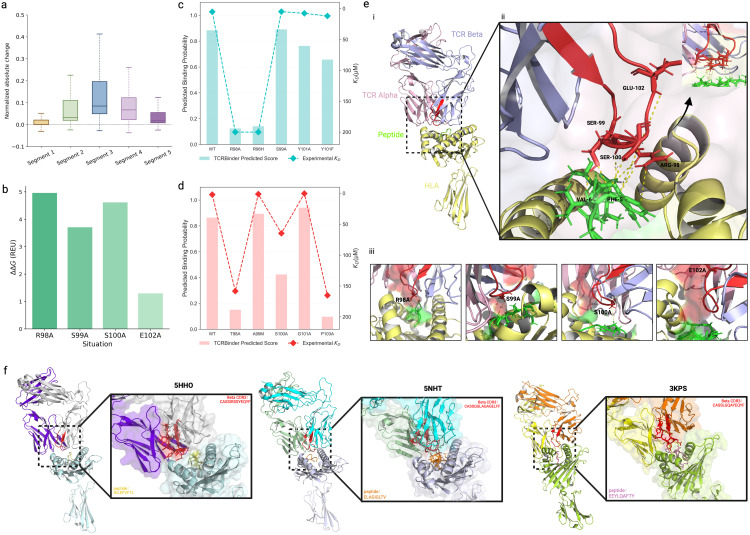
Structural and energetic analysis of pHLA-TCR recognition. **(a)** Analysis of TCRBinder score perturbation across five segments of the CDR3β loop. **(b)** Calculated change in binding free energy (ΔΔG) upon *in silico* alanine scanning of key residues in the 1OGA complex. **(c-d)** Concordance analysis between predicted binding scores and experimental affinity for JM22 (c) and A6 (d) TCR variants. **(e)** Structure of the 1OGA PHT complex (i) with a magnified view of the interaction interface (ii) and structural details for each mutation site, i.e., R98A, S99A, S100A, and E102A (iii). **(f)** Structural visualizations of three additional PHT complexes (PDB IDs: 5HHO, 5NHT, 3KPS). Created in BioRender. Li, X. (2026) https://BioRender.com/px2pbv5.

To systematically investigate the predictive mechanisms of the TCRBinder model and precisely pinpoint key amino acid residues that regulate TCR recognition, we integrated computational alanine scanning with structure-based analysis. We began by dividing the CDR3 region of the TCR β chain into five segments and analyzed the changes in model scores resulting from site directed alanine mutations within each segment. As illustrated in Fig 4a, the most significant score changes were concentrated in the central portions of the CDR3β chain (segments 3 and 4), while the terminal segments exhibited minimal impact. This finding is consistent with classical structural immunology principles, which indicate that the central region of the peptide typically protrudes towards the TCR, and that the apex region of the CDR3β chain interacts with this protruding segment. Consequently, residues located within this core region exert a decisive influence on recognition specificity and affinity.

To further validate this pattern and quantify the contribution of critical residues, we employed the HLA-A02:01-GILGFVFTL-TCR complex (PDB ID: 1OGA) as a specific case study, performing detailed *in silico* alanine scanning using Rosetta software. We sequentially substituted candidate residues on both the CDR3β and the peptide with alanine and calculated the resulting changes in binding free energy (ΔΔG). The results presented in Fig 4b clearly delineate an energy importance hierarchy, which shows that the model-predicted key residues R98, S99, and S100, when substituted with alanine (R98A, S99A, S100A), all induced significant destabilizing effects (i.e., large ΔΔG values). In contrast, the mutation at the non-critical site, E102A, resulted in only a negligible loss of energy. These energetic trends not only confirm the attribution accuracy of the TCRBinder model but also unequivocally designate the R98, S99, S100 combination as a “hotspot triplet” situated at the apex of CDR3β loop.

Beyond binding energy analysis, we conducted structural analysis to further elucidate the physical basis for these energetic differences. The complete 1OGA ternary complex and its interface contact network are presented in Fig 4e. Residues R98, S99, and S100 at the CDR3β apex are positioned in close proximity to the peptide’s center and the HLA α-helical groove, forming a dense network of close-range contacts. Following the substitution of these key residues with alanine, the shortest distances between the peptide and TCR generally increased, leading to a reduction in interface contact area. Taking the S99A mutation as an example, the shortest hydrogen bond distance between TCR-S99 and peptide-F5 increased significantly from 2.5 Å to 4.0 Å, whereas the geometric changes induced by the non-core site TCR-E102 mutation were considerably smaller. This geometric alteration is consistent with the ΔΔG ranking in Fig 4b, supporting the critical role of these residues in interface stability. Finally, to test the generalizability of this finding, we applied this analytical framework to three additional complexes with distinct peptides and HLA alleles (PDB IDs: 5HHO, 5NHT, 3KPS). As shown in Fig 4f, we observed similar patterns dominated by recognition from the central region of the CDR3β, demonstrating the reliability and universality of this analytical approach in identifying core PHT interaction patterns.

To further validate the model’s interpretability against ground-truth experimental data beyond theoretical energy calculations, we benchmarked TCRBinder using deep mutational scanning records retrieved from the TRAIT database [48]. We analyzed two distinct PHT complexes with extensive mutational profiles: the JM22 TCR targeting the Influenza A epitope (GILGFVFTL) and the A6 TCR targeting the HTLV-1 Tax epitope (LLFGYPVYV). By comparing predicted binding probabilities with experimentally measured dissociation constants ($K_{D}$), we observed a strong concordance between model outputs and biological affinity (Fig 4c and 4d). Specifically, TCRBinder correctly identified experimentally validated hotspots, such as residue R98 in JM22 and residues T98/P103 in A6, where mutations resulted in a complete loss of binding and a corresponding sharp drop in predicted scores. Notably, the model also exhibited sensitivity to affinity-enhancing mutations; for the A6 TCR, it assigned the highest probability to the G101A variant, consistent with its experimentally superior affinity ($K_{D}$=0.33μM) compared to the wild type ($K_{D}$=2.11μM). This validation confirms that TCRBinder effectively captures critical physicochemical dependencies and residue-level determinants of specificity.

## Discussion

We present TCRBinder, a unified model that integrates a pre-trained language model with a paired chain consistency predictor to predict antigen specificity by learning joint representations of paired TCR α and β chains. Its architecture captures binding determinants that may be overlooked by single-chain models by leveraging the joint context of both TCR chains during training. Our results demonstrate that this paired chain approach achieves state-of-the-art predictive performance on PHT binding tasks, outperforming models that consider only the TCR β chain or employ separate encoders. By pre-training on large-scale TCR repertoire data, TCRBinder incorporates both generalizable sequence patterns and less common motifs into its embeddings, significantly improving downstream predictive accuracy compared to traditional clustering or motif-based methods. These findings highlight the architectural advantages of integrating paired chain information and validate the benefits of fully considering both TCR α and TCR β chain sequences for binding specificity.

The substantial improvement in TCRBinder’s performance aligns with emerging evidence that deep learning and language model embeddings can surpass the accuracy of earlier pTCR prediction tools. Our unified design, which reinforces inter-chain interactions, yields a richer representation of the TCR interface. In benchmark comparisons, this approach demonstrates enhanced generalization, likely because the model captures paired chain synergy and subtle inter-chain dependencies that are lost when processing the chains independently. The inclusion of both chains aligns with the biological reality of the TCR α and β chains acting as a single recognition unit and is consistent with previous findings where incorporating TCR α chain data significantly boosted performance [16,49]. By leveraging a pre-trained language model, TCRBinder can identify biochemically relevant sequence features, such as conserved motifs or physicochemical preferences, similar to recent TCR-specific language models that capture amino acid properties and positional biases. To illustrate TCRBinder’s practical potential, we conducted in-depth analyses, including identifying TCR clusters specific to certain antigens and estimating SARS-CoV-2 spike and non-spike specific T-cell responses. These strengths underscore the methodological advances of TCRBinder over approaches based purely on sequence similarity or shallow machine learning.

Despite these advances, limitations in structural modeling remain. The antigenic peptide and HLA are encoded by separate ESM2 modules, which overlooks the biophysical coupling inherent in pHLA complex formation. Future work will address this by adopting a paired pHLA encoder or pre-training strategy. TCRBinder currently relies solely on TCR specificity labels and lacks direct supervision from pHLA binding data. Integrating these presentation priors could enhance predictive robustness, particularly for neoantigens with low HLA binding affinity.

## Materials and methods

### Datasets

The scarcity of positive PHT binding data often necessitates data aggregation from multiple sources to build a more substantial dataset for training and evaluation. We collected positive binding triples for training from four publicly available datasets: VDJdb [30], IEDB [31], OTS [50], and McPAS-TCR [51]. In light of recent reports indicating that raw high-throughput sequencing data from 10x Genomics may contain noise and false positives [37], we adopted a rigorous data processing strategy. Specifically, we excluded raw 10x Genomics data and instead utilized a curated dataset processed by the iTRAP framework [52], which effectively eliminates sequencing artifacts. To ensure data quality, we removed all entries containing duplicate records or anomalous sequences (including missing residues or ambiguous amino acid codes). The final curated positive dataset contained 12,827 observations, covering 174 unique peptides and 23 HLA subtypes. The final dataset was randomly partitioned into training, validation, and benchmark test sets in a 3:1:1 ratio. This benchmark test set serves as the primary internal standard for comparing model performance. To construct the negative dataset, we adopted an internal mismatching strategy with a 1:5 positive-to-negative ratio. Specifically, for each positive triple, we fixed the peptide (and its associated HLA allele) and paired it with five TCRs randomly sampled from the training dataset that were originally known to bind different peptides. To minimize the risk of potential cross-reactivity (false negatives), we ensured that the sampled TCRs were derived from peptides with low sequence similarity (Levenshtein distance>3) to the target peptide.

For external validation, we retrieved two independent test sets from the VDJdb database, subject to rigorous quality control, to evaluate (1) TCR binding to unseen epitopes and (2) binding specificity across different TCR populations. Furthermore, to assess performance in a clinically relevant context, we extracted PHT binding pairs from public datasets derived from T cells of SARS-CoV-2 infected patients and uninfected individuals, which were identified using viral-derived pHLA multimers. Detailed statistics for all datasets used in this study are provided in S6 Table. Crucially, to ensure rigorous independent evaluation and prevent data leakage, any PHT interactions present in these external validation sets or the SARS-CoV-2 dataset were strictly excluded from the construction of the training and benchmark test datasets.

### Model architecture

In TCRBinder, we adopt a unified architecture that couples global context from protein language models with explicit local interaction modeling to predict PHT or pTCR binding. The antigenic peptide and HLA alleles are encoded independently by ESM2, providing evolution-aware token embeddings that capture biochemical and positional regularities. Specifically, we represent each HLA allele by a fixed-length pseudo-sequence consisting of 34 amino acid residues that contact the peptide-binding cleft. We define the contact residues as positions within 4.0 Å of the peptide in representative HLA-A and HLA-B structures with nonamer peptides. We include polymorphic residues from the HLA-A and HLA-B loci at these contact positions, yielding a 34-residue pseudo-sequence that is used as the ESM2 input for HLA [53]. The paired TCR α and β chains are encoded by two Roformer encoders that are pre-trained with masked language modeling on unlabeled TCR repertoires, using rotary positional embeddings to preserve order and long-range dependencies while learning chain-specific grammar. The four sequence embeddings are processed in a branch-wise manner by the MFCNN, where each branch applies multi-scale 1D convolutions to its corresponding input to extract local sequence patterns. The resulting four branch features are then aligned and concatenated to form a joint feature, which is subsequently fed into the MLP prediction head to produce the final binding specificity. In addition, residual connections, layer normalization, and dropout are used to stabilize optimization and enhance generalization, while a reshape layer combined with pointwise fully connected mixing is employed to integrate feature-map channels within each branch into compact feature maps. The MFCNN therefore aggregates short- and medium-range contact patterns while remaining computationally lightweight and robust to sequence length variation. Its outputs are summarized by global max pooling and fed to an MLP layer that produces the final prediction. This end-to-end design leverages pre-trained encoders for global semantics and the MFCNN for localized sequence motifs extracted in each branch, which together yield accurate predictions across diverse antigens and HLA alleles and support interpretation through activation-based attributions that highlight salient residues at the PHT interface. The detailed hyper-parameters of the PHT binding prediction model are presented in S7 Table.

### Pre-training and task-specific adaptation of TCRBinder

In both the large-scale pre-training and the downstream fine-tuning phases, we employ a unique amino acid (UAA) tokenizer tailored to the single residue sensitivity of T-cell receptor (TCR) sequences. Under the UAA scheme, every amino acid residue present in the corpus was assigned an exclusive token index, thereby establishing a strict one-to-one mapping between sequence characters and model inputs. Each token was encoded as an integer and subsequently transformed into a continuous vector representation through a trainable embedding matrix. This encoding approach allows the preservation of single-residue substitutions and small indels while maintaining sensitivity to positional context that is essential for capturing the subtle sequence variations that frequently modulate TCR recognition. Pre-training was conducted under the masked amino acid (MAA) paradigm, analogous to BERT’s masked language modeling objective. Specifically, 15% of residues were randomly substituted with a special [mask] token, and the model was tasked with recovering the masked amino acids using only surrounding context, thereby enabling the network to learn local biochemical cues together with long-range structural dependencies in a fully self-supervised fashion. To encode paired chain TCR sequences, we adopted a Transformer-based backbone consisting of 12 stacked Roformer blocks, each equipped with 12 attention heads, a hidden size of 640, and a feed forward dimension of 2560, with GeLU activation applied to hidden layers. Training was performed in 32-bit floating point precision (FP32) to ensure numerical stability. Both TCR α and β chain models were trained on four NVIDIA GeForce A100 GPUs with data parallelism. In the pre-training, over 5.3 million TCR sequences were used to optimize the α-Roformer and β-Roformer encoders. We employed the AdamW optimizer with a learning rate of 5e-5, a warmup ratio of 0.1, and the training took approximately 20 hours. The detailed hyper-parameters of the pre-training process are presented in S8 Table.

### ESM2-based protein representation

To obtain high-quality protein sequence representations for antigenic peptides and HLA pseudo-sequences, we employed the large protein language model ESM2–150. It takes raw amino acid sequences as input and generates residue-level embeddings through a transformer-based architecture pretrained with a masked language modeling objective. In this work, we utilized the embedding vectors from the final hidden layer of ESM2 to characterize each amino acid position in the sequence.

We used the publicly available 150 million parameter ESM2 model for all experiments. The ESM2 model was implemented via the HuggingFace Transformers library, and the final protein embedding for each input was obtained as the output of the respective pooling strategy. This approach enables the seamless integration of evolutionary and structural information embedded in ESM2, providing highly informative features for both PHT and pTCR binding prediction tasks in TCRBinder.

### Roformer for TCR sequence representation

The Roformer encoder is employed to capture the long-range dependencies and relative positions within TCR α and β chain sequences by leveraging rotary position embeddings (RoPE). This mechanism applies a learnable rotation to each amino acid’s embedding vector according to its position, facilitating the modeling of sequence order and spatial information. Suppose the input TCR sequence of length *L* is represented as:


$$\begin{array}{c} {𝒵}_{ℒ}=\{h_{p}{\}}_{p=1}^{L} \end{array}$$ (1)

where $h_{p}$ is the embedding vector for the p-th amino acid residue. The self-attention mechanism constructs queries, keys, and values as follows:


$$\begin{array}{c} Q_{p}=\psi_{q}(h_{p},p),\;K_{q}=\psi_{k}(h_{q},q),\;V_{q}=\psi_{v}(h_{q},q) \end{array}$$ (2)

where $\psi_{q}()$, $\psi_{k}()$ and $\psi_{v}()$ are learnable mappings that combine sequence and positional information. The attention weights between positions *p* and *q* are computed as:


$$\begin{array}{c} \alpha_{p,q}=\frac{\exp\;\left(\frac{Q_{p}^{⊤}K_{q}}{\sqrt{d_{k}}}\right)}{\sum_{t=1}^{L}\exp\;\left(\frac{Q_{p}^{⊤}K_{t}}{\sqrt{d_{k}}}\right)} \end{array}$$ (3)

where $d_{k}$ is the dimensionality of the key vectors. To encode relative positions, RoPE rotates the projected embeddings according to position:


$$\begin{array}{c} \psi_{q}(h_{p},p)=(M_{q}h_{p})\cdot rot(p),\;\psi_{k}(h_{q},q)=(M_{k}h_{q})\cdot rot(q) \end{array}$$ (4)

where $M_{q}$ and $M_{k}$ are trainable matrices, and rot() denotes a positional rotation operator (sinusoidal transformation). The attention computation therefore encodes the relative position as:


$$\begin{array}{c} \left\langle \psi_{q}(h_{p},p),\;\psi_{k}(h_{q},q)\right\rangle =\phi (h_{p},\;h_{q},\;p-q) \end{array}$$ (5)

where ϕ() is a function that incorporates both the content of the amino acids and their relative distance in the sequence. This approach allows the model to infer both sequential and structural relationships within TCRs. For TCRBinder, independent Roformer encoders are used for TCR α and β chains, and the final representation for each chain is obtained by mean pooling the output of the last hidden layer. Only the final layer parameters are fine-tuned during training to balance adaptation and generalization.

### Convolutional fusion and discriminative prediction

Following the generation of modality-specific embeddings from the four pre-trained encoders, the final discriminative stage of TCRBinder is orchestrated by a dedicated fusion architecture designed to model the complex inter-molecular interactions. Each of the four high-dimensional representations, which are derived from the antigen, HLA, TCR α chain, and TCR β chain, is independently processed by a MFCNN module. This module is engineered to extract salient local motifs and multi-scale features from the token-level embeddings. The core of each MFCNN stream is a sequence of three identical convolutional blocks. A single block’s operation on an input tensor Z is defined as:


$$\begin{array}{c} CNN_{\text{Block}}(Z)=MaxPool(GLU(Conv1D(Z))) \end{array}$$ (6)

where *Conv*1*D*() represents a one-dimensional convolution, *GLU*() is the Gated Linear Unit activation function, and *MaxPool*() is a max pooling layer. The full multi-scale feature extraction for a given sequence, such as the antigenic peptide $\left(x^{p}\right)$ and TCR α chain $\left(x^{\alpha }\right)$, is a composition of these blocks:


$$\begin{array}{c} F_{p}=CNN_{\left(3\right)}\;(ESM2(x^{p})) \end{array}$$ (7)

$$\begin{array}{c} F_{p}^{\prime }=FCN_{\left(2\right)}(F_{p})+F_{p} \end{array}$$ (8)

$$\begin{array}{c} F_{\alpha }=CNN_{\left(3\right)}\;(Roformer(x^{\alpha })) \end{array}$$ (9)

$$\begin{array}{c} F_{\alpha }^{\prime }=FCN_{\left(2\right)}(F_{\alpha })+F_{\alpha } \end{array}$$ (10)

This hierarchical process is executed in parallel for the antigenic peptide $\left(F_{p}^{\prime }\right)$, HLA $\left(F_{h}^{\prime }\right)$, TCR α chain $\left(F_{\alpha }^{\prime }\right)$, and TCR β chain $\left(F_{\beta }^{\prime }\right)$ embeddings. Subsequently, these four refined feature maps are concatenated to form a joint, holistic feature tensor, $S_{F}$, which represents the entire quaternary complex:


$$\begin{array}{c} S_{F}=Concatenate(F_{p}^{\prime },F_{h}^{\prime },F_{\alpha }^{\prime },F_{\beta }^{\prime }) \end{array}$$ (11)

To manage the variable lengths inherent in protein sequences, the MFCNN enforces a maximum sequence length: 34 amino acids for HLA pseudo-sequences, 13 amino acids for antigenic peptides, and 130 amino acids for full-length TCR α and β chains. Inputs exceeding this predefined threshold are truncated, while shorter sequences are padded with a designated [PAD] token. This strategy ensures that all inputs are converted into fixed-size tensors, a necessary step for efficient, batched computation in deep learning frameworks.

The high-level joint feature is then passed to the final prediction head, which consists of an MLP layer. Finally, the PHT binding specificity *BS* is obtained.


$$\begin{array}{c} BS=Sig(MLP(S_{F})) \end{array}$$ (12)

where *Sig*() is the Sigmoid function.

### Key amino acid residue identification strategy

TCRBinder uses full-length paired TCR chains to evaluate binding specificity, but we further focused on the CDR3 loop of the β chain, which typically provides the primary contact with the peptide. To support our analysis and verify our findings in 3D space, we screened PHT crystal complexes (N = 112) from a previous study [29]. From these PHT complexes, we extracted CDR3, peptides, and HLA sequences. To study the role of the CDR3β loop in the binding of PHT, the CDR3 sequence was divided into 5 fragments by using the sliding window method. This method dynamically adjusted the fragment length according to the total sequence length to achieve nearly equal division, and the central fragment was given priority to cover the key contact area. For instance, given a CDR3β sequence of length 15, five segments of equal length can be divided. This segmentation was checked against structural contact information to ensure consistency with crystallographic data. Alanine substitutions were then introduced within each segment, and the modified sequences were evaluated using TCRBinder. Binding scores were summarized according to the position of the substituted residues to identify sites most critical for recognition.

## Supporting information

S1 Appendix Evaluation metrics and methods. (DOCX)

S1 Fig Performance of TCRBinder and state-of-the-art methods on the benchmark test dataset and VDJdb test dataset in PHT task. Created in BioRender. Li, X. (2026) https://BioRender.com/px2pbv5.
(TIF)

S2 Fig Comparison results of TCRBinder and state-of-the-art methods on the test dataset in the pTCR task. Created in BioRender. Li, X. (2026) https://BioRender.com/px2pbv5.
(TIF)

S3 Fig AUC-ROC and AUPR of TCRBinder, state-of-the-art methods, and control settings on the unseen test dataset. Created in BioRender. Li, X. (2026) https://BioRender.com/px2pbv5.
(TIF)

S4 Fig Performance of TCRBinder and state-of-the-art methods on the unseen test dataset and SARS-CoV-2 dataset. Created in BioRender. Li, X. (2026) https://BioRender.com/px2pbv5.
(TIF)

S5 Fig Comparison of TCRBinder with AlphaFold on the IMMREP25 benchmark. Created in BioRender. Li, X. (2026) https://BioRender.com/px2pbv5.
(TIF)

S1 Table The performance comparison between TCRBinder and other baseline models on the TCR-Antigen-HLA benchmark test dataset. (XLSX)

S2 Table The performance comparison between TCRBinder and other baseline models on the Unseen test dataset. (XLSX)

S3 Table The performance comparison between TCRBinder and other baseline models on the VDJdb test dataset. (XLSX)

S4 Table The performance comparison between TCRBinder and other baseline models on the SARS-CoV-2 dataset. (XLSX)

S5 Table The performance comparison between TCRBinder and its ablation variants on the benchmark test dataset. (XLSX)

S6 Table Statistical information of the datasets used for model training and evaluation. (XLSX)

S7 Table Detailed information of Hyper-parameters for PHT Binding Prediction. (XLSX)

S8 Table Detailed information of Hyper-parameters for Pre-training. (XLSX)
