# Supplementary material for: TCRBinder: Unified pre-trained language model with paired-chain synergy for predicting T-cell receptor binding specificity
Source: PLoS Comput Biol. 2026 Jun 22;22(6):e1014396. doi: 10.1371/journal.pcbi.1014396 (PMC13298993; doi:10.1371/journal.pcbi.1014396)
Supplement: S1 Appendix — (DOCX) [file pcbi.1014396.s001.docx]

TCRBinder: unified pre-trained language model with paired-chain synergy for predicting T-cell receptor binding specificity

Weihe Dong^1^, Qiang Yang^1,2^, Long Xu^1^, Xiaokun Li^1,3,4,5*^, Kuanquan Wang^1^, Suyu Dong^6,7,8*^, Gongning Luo^1^, Xianyu Zhang^9^, Tiansong Yang^10^, Xin Gao^6,7,8^ and Guohua Wang^1*^

^1^Faculty of Computing, Harbin Institute of Technology, Harbin, China.

^2^Zhengzhou Advanced Research Institute, Harbin Institute of Technology, Zhengzhou, China.

^3^School of Computer and Big Data, Heilongjiang University, Harbin, China.

^4^Postdoctoral Program of Heilongjiang Hengxun Technology Co., Ltd., Harbin, China.

^5^Shandong Hengxun Technology Co., Ltd., Qingdao, China.

^6^ Computer Science Program, Computer, Electrical and Mathematical Sciences and Engineering Division, King Abdullah University of Science and Technology (KAUST), Thuwal, Kingdom of Saudi Arabia.

^7^ Center of Excellence for Smart Health (KCSH), King Abdullah University of Science and Technology (KAUST), Thuwal, Kingdom of Saudi Arabia.

^8^ Center of Excellence on Generative AI, King Abdullah University of Science and Technology (KAUST), Thuwal, Kingdom of Saudi Arabia.

^9^Department of Breast Surgery, Harbin Medical University Cancer Hospital, Harbin, China.

^10^Department of Rehabilitation, the First Affiliated Hospital of Heilongjiang University of Traditional Chinese Medicine, and Traditional Chinese Medicine Informatics Key Laboratory of Heilongjiang Province, Harbin, China.

Corresponding authors: **Xiaokun Li**, Faculty of Computing, Harbin Institute of Technology, Harbin, China; E-mail: 24B303024@stu.hit.edu.cn; **Suyu Dong**, Computer Science Program, Computer, Electrical and Mathematical Sciences and Engineering Division, King Abdullah University of Science and Technology (KAUST), Thuwal, Kingdom of Saudi Arabia; E-mail: dongsuyu@126.com; **Guohua Wang**, Faculty of Computing, Harbin Institute of Technology, Harbin, China; E-mail: ghwang@hit.edu.cn.

**Section A**

In this study, we evaluated the performance of the proposed deep learning model, TCRBinder, using a comprehensive set of metrics, including the Area Under the Receiver Operating Characteristic Curve (AUC-ROC), the Area Under the Precision-Recall Curve (AUPR), Accuracy, Recall, Precision, and F1-score. These metrics provide a multifaceted assessment of the model's ability to predict T-cell receptor (TCR) binding specificity to peptide-HLA (pHLA) complexes, accounting for both overall discrimination power and handling of class imbalance in the datasets.

AUC-ROC quantifies the model's capacity to distinguish between positive and negative classes across various classification thresholds. It is computed as the area under the curve plotting the True Positive Rate (TPR, also known as Recall or Sensitivity) on the y-axis against the False Positive Rate (FPR) on the x-axis, where:

$$TPR=\frac{TP}{TP+FN}, FPR=\frac{FP}{FP+TN}$$

Here, TP denotes true positives, FN false negatives, FP false positives, and TN true negatives. A higher AUC-ROC value, approaching 1, indicates superior separation of binders from non-binders, making it particularly useful for evaluating general predictive robustness.

AUC0.1 represents the partial area under the ROC curve restricted to a False Positive Rate (FPR) range of [0, 0.1]

$$AUC0.1=\int_{0}^{0.1} \text{TPR}\left( \text{FPR} \right) d\text{FPR}$$

The term "AUC 0.1" in this work refers to the Macro-averaged AUC 0.1. Specifically, we first calculate the partial AUC independently for each peptide in the test set and then compute the arithmetic mean across all peptides:

$$\text{Macro }\text{AUC}_{0.1}=\frac{1}{N}\sum_{n=1}^{N} \text{AUC}_{0.1}(n)$$

AUPR complements AUC-ROC by focusing on the trade-off between Precision and Recall, which is especially valuable for imbalanced datasets where positive instances (e.g., binding TCR-pHLA pairs) are rare. It represents the area under the curve with Precision on the y-axis and Recall on the x-axis, defined as:

$$Precision=\frac{TP}{TP+FP}, Recall=\frac{TP}{TP+FN}$$

This metric emphasizes the model's precision in identifying true binders while maintaining recall, providing insights into performance on skewed distributions common in immunological data.

Accuracy measures the overall proportion of correct predictions, calculated as:

$$Accuracy=\frac{TP+TN}{TP+TN+FP+FN}$$

While straightforward, it can be inflated in imbalanced scenarios by favoring the majority class; thus, we interpret it alongside other metrics to ensure balanced evaluation.

Recall (or Sensitivity) assesses the model's ability to capture all actual positive cases, as given by the formula above, highlighting its sensitivity to detecting true TCR-pHLA interactions without missing many binders.

Precision evaluates the accuracy of positive predictions, per the formula above, ensuring that identified binders are reliable and minimizing false alarms.

F1-score harmonizes Precision and Recall into a single metric, computed as:

$$F1-score=2\boldsymbol{\times}\frac{Precision\boldsymbol{\times}Recall\mathbf{}}{Precision\mathbf{+}Recall\mathbf{}}$$

It is particularly effective for summarizing performance when both false positives and false negatives carry significant costs, offering a balanced view of the model's precision-recall trade-off in our TCR binding prediction tasks.

**Section B**

**STAPLER** is a transformer language model that predicts TCR-peptide specificity by processing a joint TCRαβ-peptide input[1]. It employs a two-stage learning strategy, beginning with a masked language modeling (MLM) pre-training task on large, unlabeled datasets to learn the intrinsic "grammar" of TCR and peptide sequences. This pre-trained model is then fine-tuned on labeled interaction data, allowing it to efficiently learn recognition patterns even from limited examples. By creating a shared language representation for both receptor and peptide, STAPLER effectively captures complex inter-sequence dependencies and demonstrates superior performance, particularly for antigens with sparse training data.

**NetTCR-2.2** is an enhanced deep learning framework for predicting TCR-peptide binding, built upon a convolutional neural network architecture that improves upon its predecessor[2]. It innovates by integrating a hybrid training scheme that combines a pan-specific model, trained across multiple epitopes, with fine-tuned, peptide-specific models. To address data imbalance and improve robustness, NetTCR-2.2 also incorporates adaptive loss-scaling and ensembles its predictions with a sequence similarity-based component. This multi-faceted approach allows the model to leverage shared information for peptides with sparse data while maintaining high specificity for well-characterized ones, establishing state-of-the-art performance in independent benchmarks.

**MixTCRpred** is an epitope-specific TCR-epitope interaction predictor based on a transformer encoder architecture[3]. Unlike pan-specific approaches, MixTCRpred trains a distinct model for each individual epitope, allowing it to learn highly specialized recognition rules from a large, curated dataset of paired αβTCRs targeting both class I and class II pMHCs. The model not only achieves high accuracy in predicting TCR binding for viral and cancer antigens but also serves as a powerful tool for quality control in single-cell datasets and for the functional annotation of chains in dual-alpha T cells. This specialized approach enables robust interpretation of TCR sequencing data in both bulk and epitope-specific contexts.

**EPACT** is a deep learning framework designed to predict TCR-peptide specificity by jointly encoding paired TCR chains and peptide sequences alongside MHC context[4]. Improving upon methods that rely solely on CDR3 or single chains, EPACT integrates information from multiple CDR loops to capture the full structural complexity of the binding interface. The model leverages a comprehensive representation learning strategy that processes the receptor and antigen as a unified system, allowing it to extract intricate recognition patterns from paired-chain data. By effectively modeling the non-linear dependencies between the dual-chain TCR and the pMHC, EPACT demonstrates superior generalization capabilities on diverse benchmarks, establishing a rigorous baseline for evaluating paired-chain recognition accuracy.

**TEIM** is an interpretable deep learning framework that predicts TCR-peptide binding by leveraging a transformer-based architecture with attention mechanisms[5]. Instead of treating sequences as monolithic inputs, TEIM explicitly models pairwise interactions between amino acids from the TCR and the peptide. By incorporating physicochemical properties, the model learns a biochemically informed representation of the binding interface. Its attention weights provide a direct way to visualize and interpret the key residue contacts driving specificity, offering a powerful tool for mechanistic investigation and rational TCR engineering.

**TCR-AI** is a machine learning framework specifically engineered to predict TCR-antigen specificity from high-throughput, dextramer-based screening datasets[6]. It integrates deep sequence embeddings with a robust classification architecture to model the complex relationships between TCR sequences and their cognate antigens. Designed for scalability, TCR-AI can effectively analyze large, multiplexed experimental readouts, enabling high-resolution profiling of immune repertoires and improving the accuracy of binding predictions across diverse antigenic landscapes. This makes it a valuable tool for immune monitoring and accelerating precision immunotherapy.

**ImRex** is a deep learning model that reconceptualizes TCR-epitope specificity prediction by framing it as an image classification problem[7]. It transforms TCR and peptide sequence data into a two-dimensional interaction matrix, where each element represents the potential interplay between pairs of amino acids based on their physicochemical properties. This "image" is then processed by a convolutional neural network (CNN), which learns to identify spatially conserved binding motifs analogous to feature detection in computer vision. This novel approach allows ImRex to capture structural and positional information often lost in one-dimensional sequence models, offering a unique perspective for generalizing predictions to unseen epitopes.

**Section C**

**Benchmark dataset and evaluation setting**

For the AlphaFold-based comparison in Fig. S5, we used the corresponding IMMREP25 allele-specific benchmark subsets for HLA-A02:01 and HLA-B40:01[8]. The evaluation set contains 1,000 unique TCRs distributed across 20 peptide–MHC targets, with 50 TCRs assigned to each target. For all three AlphaFold-based baselines, each TCR-pMHC pair was first assigned a model-specific confidence-derived score, namely normalized pLDDT for AF3-TCRdock, normalized interface PAE for AF2-TCRdock, and interface-focused modified ipTM for AF2-TCRmodel2. These scores were then used to rank candidate interactions, and performance was uniformly assessed using AUROC (per-peptide mean).

**AF2-TCRdock**

The AF2-TCRdock baseline was evaluated using an AlphaFold2-based docking strategy for TCR–pMHC discrimination[9]. For each candidate TCR–pMHC pair, three structural models were generated using the AlphaFold monomer model_2_ptm configuration together with docking-oriented hybrid templates, so that the prediction process was guided toward plausible ternary geometries. For each target pair, the final structure was selected according to TCR–pMHC interface-related confidence, using the predicted aligned error (PAE) across the TCR–pMHC interface as the main criterion. Classification scores were then derived from the average TCR–pMHC PAE and normalized before ranking candidate pairs and calculating per-peptide mean AUROC.

**AF2-TCRmodel2**

The AF2-TCRmodel2 baseline was evaluated with an AlphaFold-Multimer-based strategy for TCR–pMHC complex modeling[10]. For each candidate target, five structural models were generated using AlphaFold-Multimer v2.3, one from each available model weight. These candidate structures were ranked according to the model confidence score used in the TCRmodel2 framework, and the top-ranked structure was retained for downstream scoring. Classification was based on an interface-focused score derived from ipTM, specifically a modified ipTM restricted to the TCR–pMHC interface rather than to the entire multichain complex, so that the score reflected confidence in the receptor–ligand binding geometry. This interface-restricted score was used for ranking and for computing per-peptide mean AUROC.

**AF3-TCRdock**

The AF3-TCRdock baseline was evaluated using an AlphaFold3-based TCR-pMHC modeling strategy[11]. The modeling procedure incorporated inter-chain template information and docking-oriented template examples so that the relative arrangement of the TCR and pMHC was guided by prior ternary complex geometry rather than inferred solely from independent chain folding. For each candidate TCR–pMHC pair, structural predictions were generated and ranked by model confidence. The final classification score was derived from pLDDT-based confidence values and subsequently normalized across peptides and TCRs to reduce peptide-level and TCR-level confidence biases. These normalized scores were then used to rank candidate pairs and to calculate per-peptide mean AUROC.

**Reference**

1. Kwee B P Y, Messemaker M, Marcus E, et al. STAPLER: efficient learning of TCR-peptide specificity prediction from full-length TCR-peptide data. bioRxiv. 2023; 04.25:538237.
2. Jensen MF, Nielsen M. Enhancing TCR specificity predictions by combined pan- and peptide-specific training, loss-scaling, and sequence similarity integration. eLife. 2024;12:RP93934.
3. Croce G, Bobisse S, Moreno D L, et al. Deep learning predictions of TCR-epitope interactions reveal epitope-specific chains in dual alpha T cells. Nature Communications. 2024; 15: 3211.
4. Zhang Y, Wang Z, Jiang Y, et al. Epitope-anchored contrastive transfer learning for paired CD8+ T cell receptor–antigen recognition. Nature Machine Intelligence. 2024; 6: 1344–1358.
5. Peng X, Lei Y, Feng P, et al. Characterizing the interaction conformation between T-cell receptors and epitopes with deep learning. Nature Machine Intelligence. 2023;5:395-407.
6. Zhang W, Hawkins PG, He J, et al. A framework for highly multiplexed dextramer mapping and prediction of T cell receptor sequences to antigen specificity. Sci Adv. 2021;7:eabf5835.
7. Moris P, De Pauw J, Postovskaya A, et al. Current challenges for unseen-epitope TCR interaction prediction and a new perspective derived from image classification. *Brief Bioinform.* 2021;22:bbaa318.
8. Garcia Noceda M, Noakes MT, FigPope A, et al. ImmSET: Sequence-Based Predictor of TCR-pMHC Specificity at Scale. Proc Mach Learn Res. 2025;297.
9. Bradley P. Structure-based prediction of T cell receptor:peptide-MHC interactions. eLife. 2023;12:e82813.
10. Yin R, Ribeiro-Filho HV, Lin V, et al. TCRmodel2: high-resolution modeling of T cell receptor recognition using deep learning. Nucleic Acids Res. 2023;51(W1):W569-W576.
11. Visani GM, Pun MN, Minervina AA, et al. T cell receptor specificity landscape revealed through de novo peptide design. Proceedings of the National Academy of Sciences. 2025;122(42):e2504783122.
